# Screening a Peptide Library by DSC and SAXD: Comparison with the Biological Function of the Parent Proteins

**DOI:** 10.1371/journal.pone.0004356

**Published:** 2009-02-05

**Authors:** Ana J. Pérez-Berná, George Pabst, Peter Laggner, José Villalaín

**Affiliations:** 1 Instituto de Biología Molecular y Celular, Universidad “Miguel Hernández”, Alicante, Spain; 2 Institute of Biophysics and Nanosystems Research, Austrian Academy of Sciences, Graz, Austria; University of Minnesota, United States of America

## Abstract

We have recently identified the membranotropic regions of the hepatitis C virus proteins *E1*, *E2*, *core* and *p7* proteins by observing the effect of protein-derived peptide libraries on model membrane integrity. We have studied in this work the ability of selected sequences of these proteins to modulate the L_β_-L_α_ and L_α_-H_II_ phospholipid phase transitions as well as check the viability of using both DSC and SAXD to screen a protein-derived peptide library. We demonstrate that it is feasible to screen a library of peptides corresponding to one or several proteins by both SAXD and DSC. This methodological combination should allow the identification of essential regions of membrane-interacting proteins which might be implicated in the molecular mechanism of membrane fusion and/or budding.

## Introduction

Hepatitis C virus (HCV), an enveloped positive single-stranded RNA virus, is an important public health problem since it is the leading cause of acute and chronic liver disease in humans, including chronic hepatitis, cirrhosis, and hepatocellular carcinoma [1], [2], [3]. Currently, there is not vaccine to prevent the HCV infection and the available therapeutic agents, apart from being associated to different adverse effects, have very limited efficacy against the virus [4]. HCV entry into the host cell is achieved by fusion of the viral and cellular membranes, and morphogenesis and virion budding has been suggested to take place in the endoplasmic reticulum [5]. The HCV genome is widely heterogeneous, and replication errors cause a high rate of mutations [6]. The variability of the HCV proteins gives the virus the ability to escape the host immune surveillance system and notably hampers the development of an efficient vaccine. Thus, finding inhibitors of protein-membrane and protein-protein interactions involved in virus fusion and/or budding could be an alternative and valuable strategy against HCV infection since they could be potential therapeutic agents.

**Figure 1:**
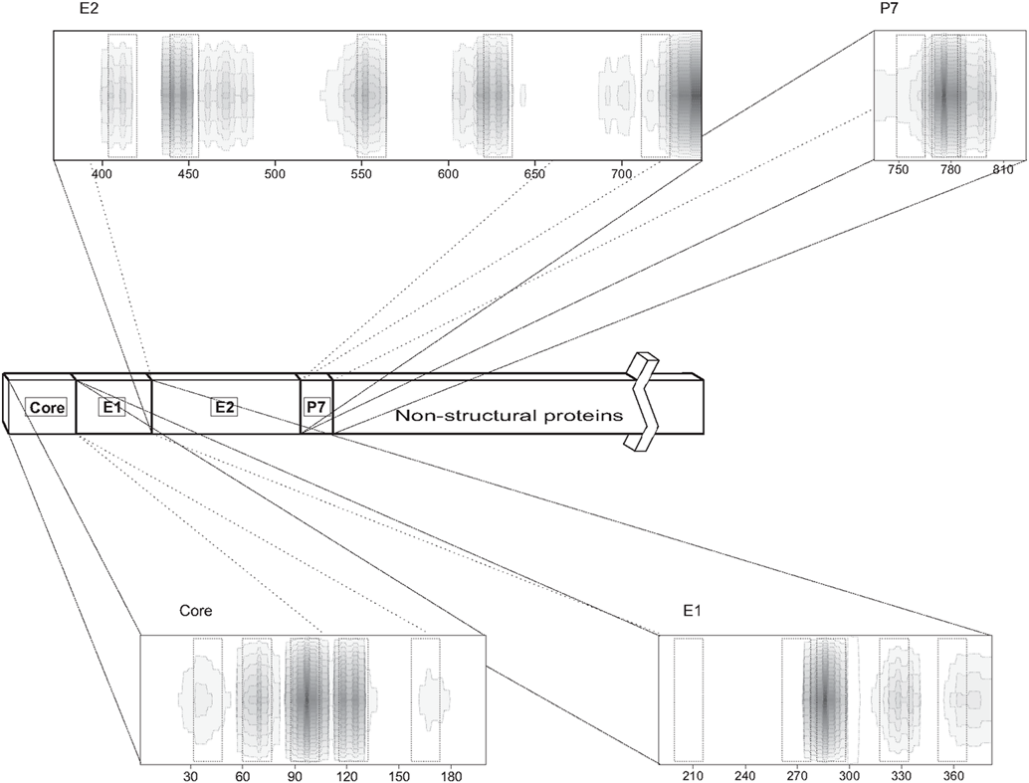
Scheme of the HCV polyprotein according to literature consensus, including the structural and non-structural proteins. The sequence and relative location of the 18-mer peptides used in this work, derived from the HCV core, E1, E2 and p7 proteins, are shown with respect to the sequence of the polyprotein. A summary of the normalized experimental membrane rupture data corresponding to 18-mer peptide libraries derived from the whole HCV core [22], E1 [23], E2 [23] and p7 [21] proteins is also shown (the darker, the higher).

The HCV genome consists of one translational open reading frame encoding a polyprotein precursor, including structural and non-structural proteins, that is cleaved by host and viral proteases (Figure 1A). The structural proteins include the protein core, which forms the viral nucleocapsid, and the envelope glycoproteins E1 and E2, both of them transmembrane proteins. The structural proteins are separated from the non-structural proteins by the short membrane protein p7. The core protein is highly basic and shows homology with the nucleocapsid protein of other flaviviruses. This protein is well conserved among the different HCV strains [7] and has regulatory roles on cell functions like immune presentation, apoptosis, lipid metabolism and transcription [8], [9], [10]. Recombinant cDNA expression studies of this protein have identified two major protein core species, p23 and p21, being the later the predominant species [9]. Significantly, the mature core protein is a dimeric α-helical protein exhibiting membrane protein features [11]. The two envelope glycoproteins, E1 and E2, are anchored to the host cell-derived double-layer lipid envelope. E1 and E2 are essential for entry, binding to receptors and inducing fusion with the host-cell membrane as well as in viral particle assembly [12]. E1 and E2 are type-I transmembrane (TM) glycoproteins, with N-terminal ectodomains and a short C-terminal TM domain. These proteins interact with each other and assemble as non-covalent heterodimers and their TMs play a major role in heterodimer formation, membrane anchoring and endoplasmic reticulum retention [13]. E1 and E2 are thought to be class II fusion proteins because the putative fusion peptide is supposedly localized in an internal sequence linked by antiparallel β-sheets [14]. Protein p7 is classified neither as structural protein nor as non-structural, locates in the cell ER and has been described to be a viroporin-like protein [15], [16], [17], [18]. p7 is essential for efficient assembly and release of infectious virions indicating that p7 is primarily involved in the late phase of the virus replication cycle [17]. At a molecular level, p7 is a small transmembrane protein with two transmembrane helical domains connected by a loop [19], [20]. One possible role for this protein could be the transport of ions from the ER to the cytoplasm of HCV-infected host cells, suggesting that p7 could be a attractive candidate for antiviral drug development.

Recently, we have identified the membrane-active regions of the HCV E1, E2, core and p7 proteins by observing the effect of protein-derived peptide libraries on model membrane integrity [21], [22], [23]. These results have permitted us to suggest the possible location of different segments in these proteins which might be implicated in protein-lipid and protein-protein interactions, helping us to understand the mechanisms underlying the interaction between viral proteins and membranes. Moreover, it has been found a good correlation between the ability of different peptides to affect the lamellar to hexagonal phase transition (L_α_→H_II_) of synthetic membranes with membrane perturbation and/or fusogenic activity [24]. Therefore we have selected different peptides from the HCV proteins E1, E2, core and p7 showing different membrano-active properties in order to screen and examine their effect on the polymorphic phase behavior of 1,2-dielaidoyl-sn-glycero-3-phosphatidylethanolamine (DEPE) by using differential scanning calorimetry (DSC) and small angle X-ray diffraction (SAXD).

## Results

The peptides used in this study and their correlation with the HCV proteins core, E1, E2 and p7 are shown in Figure 1. In our previous works [21], [22], [23], we examined the membranotropic activity of a series of peptide libraries pertaining to the full sequences of the core, E1, E2 and p7 proteins in order to detect their membranotropic regions. It is known that not all of the membrano-active segments of membrane fusion proteins might have a direct membrane role, i.e., membrane fusion [25]. Because of that, we have selected different peptides from the aforementioned peptide libraries in order to check their ability to modulate the polymorphism in DEPE vesicles. The peptides chosen pertained to sequences 29–46, 57–74, 85–102, 113–120 and 155–172 from the core protein, sequences 197–214, 260–277, 281–298, 316–333 and 351–368 from the E1 protein, sequences 400–417, 435–452, 547–564, 617–634 and 708–725 from the E2 protein and sequences 750–767, 771–788, and 785–802 from the p7 protein (see Table 1). As observed in Figure 1, several of these peptides belong to regions showing high membranotropic activities.

**Table 1:** Sequence and numbering of the peptides from the HCV proteins core, E1, E2 and p7 used in this work, as well as a summary of the experimental data.

HCV PROTEIN	PEPTIDE NUMBERING	PEPTIDE SEQUENCE	% Leakage§	% Fusion§	Uncorrelated membranes	L_α_-H_II_ disturbance*
CORE	29–46	QIVGGVYLLPRRGPRLGV	−	+	+	++
	57–74	QPRGRRQPIPKARRPEGR	+	−	−	+
	85–102	LYGNEGLGWAGWLLSPRG	+++	+	−	++
	113–130	RRRSRNLGKVIDTLTCGF	+++	−	−	−
	155–172	VRVLEDGVNYATGNLPGC	−	−	−	+++
E1	197–214	VSGIYHVTNDCSNSSIVY	−	−	−	+
	260–277	RHVDLLVGTAAFCSAMYV	+	−	−	+
	274–298	AMYVGDLCGSIFLVSQLF	+	+++	−	++
	316–333	HRMAWDMMMNWSPTTALV	+	+	+	+++
	351–368	AHWGVLAGLAYYSMVGNW	+	+	−	+
E2	400–417	TSLFSSGASQKIQLVNTN	+	+	−	+
	435–452	TGFFAALFYAHKFNSSGC	+	+	−	+
	547–564	GNWFGCTWMNSTGFTKTC	+	+	−	+
	617–634	LTPRCLVDYPYRLWHYPC	+++	+++	+	+++
	708–705	AFVSFAIKWEYILLLFLL	+	−	−	+
P7	750–767	NLVVLNAASVAGAHGILS	−	−	−	−
	771–788	FFCAAWYIKGRLAPGAAY	+++	−	−	−
	785–802	GAAYAFYGVWPLLLLLLA	+	−	−	−

§ (−), 0–15%, 15%>(+)>50%, 50%>(+++)>100% [21], [22], [23].

* (+), ΔT_HII_ between 0 and 5°C, (++) ΔT_HII_ greater than 5°C, (+++) No H_II_ phase (this work).

**Figure 2:**
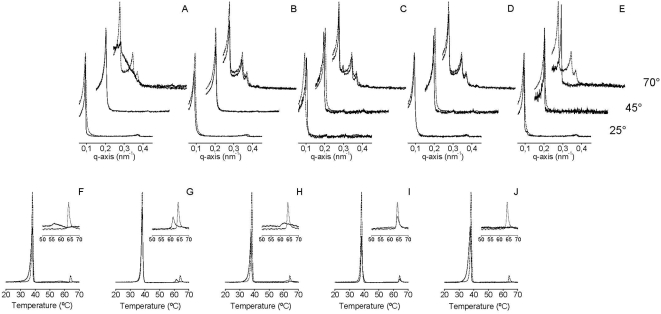
(A–E) Small angle X-ray scattering at 25°C, 45°C and 70°C and (F–J) DSC heating thermograms of DEPE in the absence (——) and in the presence (----) of the 18-mer peptides derived from the HCV core protein. The 18-mer peptides correspond to amino acid sequences 29–46 (A,F), 57–74 (B,G), 85–102 (C,H), 113–120 (D,I) and 155–172 (E,J). The peptide/phospholipid molar ratio was 1∶50 in all cases.

In order to examine the effect on these peptides on the L_β_/L_α_/H_II_ phase transitions of DEPE and obtain Information on the structural organization of the DEPE/peptide systems, we have used both DSC and SAXD [26], [27]. SAXD not only defines the macroscopic phase-structure of the phospholipid bilayer, but also provides the inter-lamellar repeat distance in the lamellar phase or the 2D-hexagonal unit cell dimensions in the hexagonal phase. Aqueous dispersions of pure DEPE undergo a gel to liquid-crystalline (L_β_→L_α_) phase transition in the lamellar phase at about 37°C and in addition a lamellar liquid-crystalline to hexagonal-H_II_ (L_α_→H_II_) phase transition at about 65°C [28]. Figure 2A shows the diffraction patterns corresponding to pure DEPE at different temperatures. Lipids organized in multilamellar structures are expected to give rise to reflections with distances which relate as 1∶1/2∶1/3 [29]. The largest first-order reflection component corresponds to the inter-lamellar repeat distance, which is comprised of the bilayer thickness and the thickness of the water layer between bilayers. Pure DEPE in the lamellar gel state at 25°C showed a characteristic first-order reflection with an inter-lamellar repeat distance of about 65 Å. As it has been previously observed in DEPE systems in the lamellar state, some higher-order reflections were not found [30]. At 45°C, pure DEPE in the fluid lamellar state showed a first-order reflection with an inter-lamellar repeat distance of about 54 Å, showing a decrease in the first-order repeat distance of about 10 Å, due to the decrease of the effective acyl chain length. Lipids organized in the hexagonal H_II_ phase give rise to reflections which relate as 1∶1/√3∶1/√4∶1/√7 [29]. In our case, pure DEPE in the hexagonal H_II_ phase at 70°C clearly showed the reflections characteristic of this phase, the first one at about 66 Å, as observed in Figure 2A. By DSC, pure DEPE presented a highly cooperative endothermic gel to liquid-crystalline phase transition T_m_ (temperature of the gel-to–liquid crystalline phase transition) at 37.8°C and a lamellar-to-inverted hexagonal H_II_ transition at 64.3°C as shown in Figure 2F. Therefore, by using both DSC and SAXD we are able of characterizing the phase polymorphism of DEPE in the presence of the chosen peptides.

Recently, we have found that the HCV core protein presents several membranotropic regions which might play an important role in its biological action, specifically on the budding process [22]. We have chosen different peptides pertaining to these membranotropic regions and studied their modulatory effect on DEPE membranes as shown in Figure 2 (sequences 29–46, 57–74, 85–102, 113–120 and 155–172). As observed in Figure 2A, the peptide corresponding to sequence 29–46 from the HCV core protein did not show any effect on the DEPE SAXD reflections at both 25°C and 45°C. However, at 70°C, the presence of a broad SAXD signal indicates the presence of uncorrelated membranes, [31] and at the same time accompanied by small reflections corresponding to the H_II_ phase. DSC indicates that the peptide decreases slightly the enthalpy of the main transition with a concomitant broadening (Figure 2F). However, it decreased significantly the enthalpy and the transition temperature of the lamellar to hexagonal phase transition of the phospholipid (from 64.2°C to 56.3°C) (Figure 2F). Peptide 57–74 did not give rise to any different SAXD reflections, showing that its interaction with the membrane did not have any significant changes in the membrane structure of DEPE (Figure 2B). By DSC, a small decrease in T_H_ was observed from 64.2°C to 61.4°C (Figure 2G). The GAP analysis of the effect produced by the peptide corresponding to sequence 85–102 shows that at 25°C the membrane thickness is slightly decreased but increased at 45°C (Figure 2C). Interestingly, at 70°C it is observed that there is a coexistence of lamellar and hexagonal phases (Figure 2C). Similarly to what was found with peptide 29–46, DSC shows that sequence 85–102 decreases slightly the enthalpy of the main transition as well as decreases the enthalpy and temperature of the T_H_ transition from 64.2°C to 62.1°C, broadening it (Figure 2H). Peptide 113–120 did not give rise to any different SAXD reflections, showing that its interaction with the membrane did not have any significant changes in the membrane structure of DEPE (Figure 2D). However, it decreases the membrane thickness at 45°C. DSC shows that the peptide decreases slightly the enthalpy of both DEPE transitions, the gel –to-liquid-crystalline transition and the lamellar-to-hexagonal phase transition, without a significant change in the transition temperatures (Figure 2I). In contrast to what was observed above, peptide 155–172 induced significant changes in the SAXD pattern of DEPE (Figure 2E). Whereas at 25°C and 45°C there were no significant differences with pure DEPE, but a small increase in membrane thickness at 25°C; at 70°C there was no signal corresponding to the hexagonal phase (Figure 2E). These results were corroborated by DSC, since the peptide, apart from decreasing the enthalpy of the main transition of the phospholipid, abolished completely the appearance of the hexagonal phase transition endotherm (Figure 2J).

**Figure 3:**
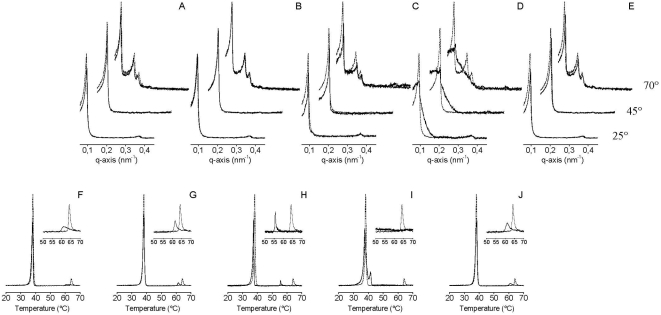
(A–E) Small angle X-ray scattering at 25°C, 45°C and 70°C and (F–J) DSC heating thermograms of DEPE in the absence (——) and in the presence (----) of the 18-mer peptides derived from the HCV E1 protein. The 18-mer peptides correspond to amino acid sequences 197–214 (A,F), 260–277 (B,G), 281–298 (C,H), 316–333 (D,I) and 351–368 (E,J). The peptide/phospholipid molar ratio was 1∶50 in all cases.

Similarly to what was found with the HCV core protein, we previously found that the HCV E1 and E2 proteins present several membranotropic regions [23]. Being E1 and E2 the proteins which are responsible of the entry of the HCV virus into the target cell, these membranotropic regions play an essential role in the biological action of these proteins. The peptides we have chosen to study their effect on DEPE are the peptides corresponding to amino acid sequences 197–214, 260–277, 281–298, 316–333 and 351–368 from the E1 protein, and 400–417, 435–452, 547–564, 617–634 and 708–725 from the E2 protein (see Figure 3).

Peptides 197–214 and 260–277 from E1 did not give rise to any significant differences in DEPE SAXD reflections, showing that its interaction with the membrane did not induce any significant changes in the membrane structure of DEPE (Figures 3A and 3B). DSC shows that the peptides decreased only slightly the enthalpy of both L_β_→L_α_ and L_α_→H_II_ transitions of DEPE, decreasing the transition temperature of the Lα-H_II_ phase transition from about 64.2°C to 61.1–61.4°C (Figures 2F and 2G). The peptide corresponding to sequence 281–298 of the E1 protein decreases the membrane thickness at both 25°C and 45°C but no significant differences were found at 70°C when compared with the pure phospholipid (Figure 3C). Interestingly, DSC shows the existence of a slight decrease in the enthalpy of both transitions of DEPE but a significant decrease in the Lα-H_II_ transition temperature from 64.2°C to 55.64°C (Figure 2H). Significantly, peptide corresponding to sequence 316–333 from HCV E1 induced a disordering (presence of uncorrelated membranes) on DEPE at all studied temperatures, as seen by SAXD (Figure 3D). Although there is an increase in the membrane thickness, the reflections pertaining to the different DEPE phases are recognized (Figure 3D). As observed by DSC, the peptide induced the disappearance of the Lα-H_II_ transition as well as it induced the appearance of another peak at about 41.51°C (Figure 3I). Similarly to peptides 197–214 and 260–277, peptide 351–368 did not give rise to any significant differences in the DEPE SAXD reflections (Figure 3E). As observed by DSC, the peptides decreased only slightly the enthalpy of both DEPE transitions, decreasing the phase transition temperature of the Lα-H_II_ transition from 64.2°C to 61.1°C (Figure 2J).

**Figure 4:**
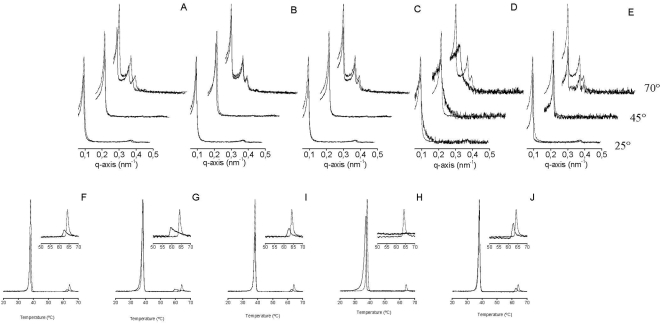
(A–E) Small angle X-ray scattering at 25°C, 45°C and 70°C and (F–J) DSC heating thermograms of DEPE in the absence (——) and in the presence (----) of the 18-mer peptides derived from the HCV E2 protein. The 18-mer peptides correspond to amino acid sequences 400–417 (A,F), 435–452 (B,G), 547–564 (C,H), 617–634 (D,I) and 708–725 (E,J). The peptide/phospholipid molar ratio was 1∶50 in all cases.

Peptides 400–417, 435–452 and 547–564 from the HCV E2 protein did not give rise to any significant differences in the DEPE SAXD reflections, showing that its interaction with the membrane did not have any significant changes in the membrane structure of DEPE (Figures 4A, 4B and 4C). As observed by DSC, the peptides induced a slight decrease in the enthalpy and temperature of the Lα-H_II_ transition (from 64.2°C to 62.5, 59.5 and 62.6°C, respectively) (Figures 4F, 4G and 4I). In contrast to these peptides, the peptide corresponding to sequence 617–634 of E2 induced a disordering on the DEPE membranes at all studied temperatures (presence of uncorrelated membranes), as observed by SAXD (Figure 4D). There is an increase in the membrane thickness at all temperatures and the reflections pertaining to the H_II_ phase of DEPE are not observed at 70°C (Figure 4D). As observed by DSC, the peptide induced the disappearance of the L_α_-H_II_ endotherm (Figure 4H). Peptide 708–725 did not give rise to any significant differences in the DEPE SAXD reflections (except for a decrease in the reflection intensity, see Figure 4E). As observed by DSC, the peptide did not induce any significant changes in the DEPE main transition but a slight decrease in the temperature of the L_α_-H_II_ transition (from 64.2°C to 62.6°C) (Figure 4J).

**Figure 5:**
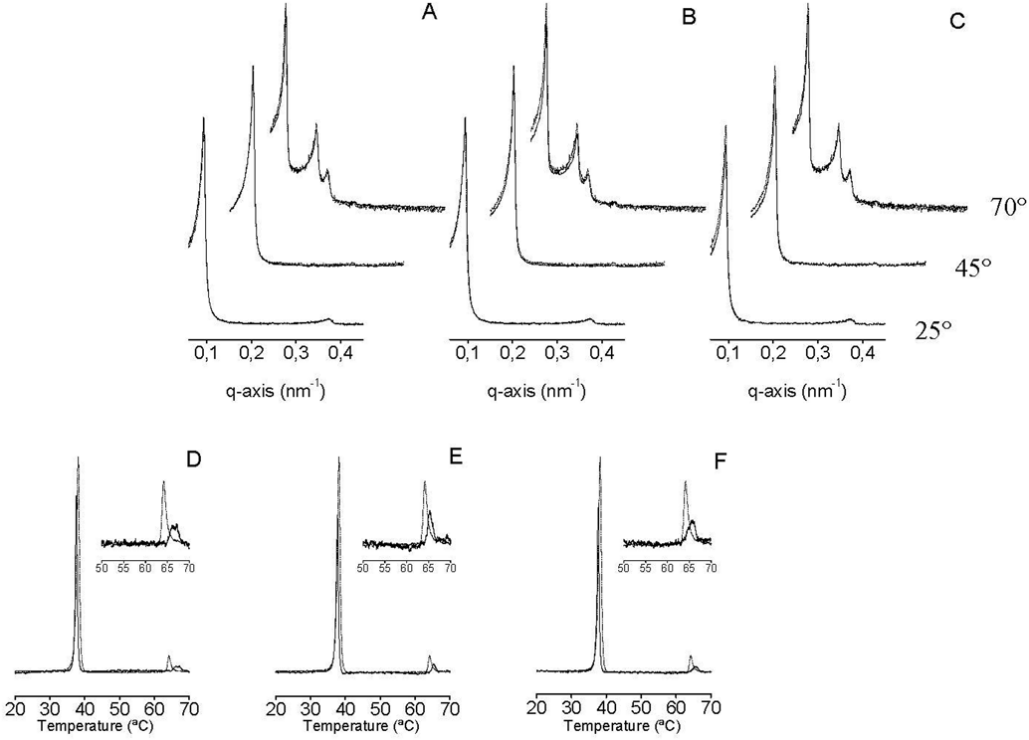
(A–C) Small angle X-ray scattering at 25°C, 45°C and 70°C and (D–F) DSC heating thermograms of DEPE in the absence (——) and in the presence (----) of the 18-mer peptides derived from the HCV E2 protein. The 18-mer peptides correspond to amino acid sequences 750–767 (A,D), 771–788 (B,E), 785–802 (C,F). The peptide/phospholipid molar ratio was 1∶50 in all cases.

We have recently identified that the HCV p7 protein presents a highly membranotropic region coincidental with the loop region [21]. However, this membranotropic region is not related to any effect on the phase polymorphism of the membrane, but to its action as a viroporin-like protein [21]. We have studied three peptides from p7 with DEPE membranes; these sequences were 750–767, 771–788, and 785–802 (Figure 5). As observed in Figure 5, peptides pertaining to sequences750–767, 771–788, and 785–802 did not give rise to any significant differences in the DEPE SAXD reflections, showing that its interaction with the membrane did not have any significant changes in the membrane structure of DEPE (Figures 5A, 5B and 5C). Interestingly, all peptides are able of decreasing the enthalpy of both DEPE transitions, the L_β_-L_α_ and L_α_-H_II_ transitions, as well as increasing the L_α_-H_II_ phase transition temperature (from 64.2°C to about 66.1–67.1, 65.3 and 65.7°C, respectively) (Figures 5D, 5E and 5F). Interestingly, peptide pertaining to sequence 750–767 is capable of inducing a broad L_α_-H_II_ phase transition which seems to have two different transitions (Figure 5D).

## Discussion

Envelope fusion glycoproteins located on the outer surface of the viral membranes mediate the fusion of the viral and cellular membranes [32], [33]. The three-dimensional structure of class I and class II membrane fusion proteins is different but their function is identical, so they must share structural and functional characteristics in specific domains which interact with and disrupt biological membranes [32], [33]. Additionally, different membrane binding regions from membrane fusion proteins are necessary to complete the fusion process [34]. In general, fusion implies the insertion of specific regions that disrupt the membrane, leading to the formation of a initial local lipid connection (lipid stalk) [35], [36]. This lipid stalk is then believed to expand into a hemifusion diaphragm whose rupture would finally generate a fusion pore [35], [36]. Membrane fusion does not necessarily occur at the plasma membrane level; viral entry can also involve endocytosis and vesicular trafficking, as it happens in the case of HCV. Moreover, one of the most significant points in the HCV viral cycle is the formation of a replication complex, composed of the viral proteins, RNA and altered membranes in infected hepatocytes forming the so called membraneous web [37], [38]. Since we have shown previously the existence of membranotropic regions of the HCV core, E1, E2 and p7 proteins [21], [22], [23], in this work we have made a specific screening for studying the ability of selected sequences of these proteins to modulate the well known L_β_-L_α_ and L_α_-H_II_ transitions of DEPE [28], with the objectives of checking a) the correlation of their activity with the propensity to modulate the polymorphic phase behavior of DEPE and b) the feasibility of using both DSC and SAXD to screen the effect of a series of peptide libraries on the polymorphic phase behavior of DEPE [25].

Protein sequences might have hydrophobic regions which form the hydrofobic core needed for protein folding. Peptides originated from these regions might interact with membranes and show membrane leakage. However, membrane perturbation by itself is not sufficient to complete the process of viral and cellular membrane fusion, since the energetic barrier for hemifusion and fusion is larger than for leakage (pore formation). Fusion peptides show generally a high membrane-perturbing and fusogenic activity, i.e., mixing membrane lipids and aqueous contents of the vesicles (low leakage and high fusion values). However, there is not a strict quantitative criterion for the degree of membrane destabilization expected for a fusion peptide. On the other hand a common property of fusion peptides is the tendency to promote negative curvatures in the membrane. For this reason fusion peptides tend to lower the bilayer to hexagonal phase transition temperature of phosphatidylethanolamines. In principle only peptides which present a significant effect on the polymorphic phase behavior of DEPE could be located in regions implicated in a stabilization/destabilization role of lamellar/non-lamellar structures, roles needed for fusion and budding.

Of the different peptides studied in this work, only peptides corresponding to sequences 29–46 (core), 316–333 (E1) and 617–634 (E2) gave rise to positionally uncorrelated membranes at high temperatures. All these peptides affected significatively the Lα-H_II_ phase transition of DEPE as well as they presented a significant effect on membrane fusion (Table 1). Apart from these peptides, other peptides which presented a significant effect on the L_α_-H_II_ phase transition of DEPE, were found to induce both membrane fusion and leakage (Table 1). Interestingly, peptide 155–172 pertaining to the core protein, which did not show any significant effect on both membrane rupture and fusion, affected significantly the hexagonal transition of DEPE. This anomalous behavior might be due to the fact that this peptide overlaps with part of the signal sequence of the E1 protein.

Another important event in viral life is morphogenesis. This step involves lipid membranes and it is not as well characterized as the fusion event. For enveloped viruses, the assembly of the virions takes place in the host cell membrane. In the case of HCV it has been suggested that the assembly of the virus particles occurs in the endoplasmic reticulum membranes where the core protein might play a central role in viral particle formation as well as it might drive the budding process [39]. Significantly, the HCV core protein is very well conserved among different HCV strains and has important regulatory roles on different cell functions. Peptide 29–46 corresponding to the core protein presents a significant effect on DEPE polymorphism and therefore could be implicated in the stabilization of non-lamellar structures needed for the budding process. E1 and E2 envelope proteins induce the fusion between the viral envelope and the host cell membrane. As observed in Table 1, peptides 316–333 from E1 and 617–634 from E2 affect significantly the polymorphic phase behavior of DEPE, induce the presence of uncorrelated membranes, and present a high effect of membrane rupture and fusion. As suggested previously, the regions were these two peptides reside could hold a similar function in the corresponding proteins as that observed for other ones (i.e., gp41). Protein p7 is classified neither as a structural nor as a non-structural protein, is located in the cell ER, and forms a membrane pore. We have recently shown the presence of a highly membranotropic region in p7 presenting high leakage but low fusion (sequence 771–788). As shown in Table 1, neither this sequence nor the other two sequences derived from p7 studied here present any significant effect on the polymorphic phase behavior of DEPE. This data would suggest that p7, although having a high membranotropic region, would be implicated neither in membrane fusion nor in budding.

In conclusion, we have demonstrated that is feasible to screen a library of peptides corresponding to one or several proteins by both SAXD and DSC, or preferably by a simultaneous combination of the two [31]. This methodology should allow the identification of those peptides which lead to the loss of lipid multilayer correlation as well as the modulation of the Lα-H_II_ phase transition, to correlate the data with experimentally determined membrane rupture and fusion, and most important, the identification of important regions of membrane-interacting proteins which might be implicated in the molecular mechanism of membrane fusion and/or budding.

## Materials and Methods

### Materials and Reagents

1,2-Dielaidoyl-sn-glycero-3-phosphatidylethanolamine (DEPE) was purchased from Avanti Polar Lipids (Alabaster, AL, USA). 18-mer peptides derived from the core protein (5 peptides), the E1 envelope glycoprotein (6 peptides), the E2 envelope glycoprotein (5 peptides) and the p7 protein (3 peptides) from hepatitis C virus strains 1B4J were obtained through the NIH AIDS Research and Reference Reagent Program (Division of AIDS, NIAID, NIH, Bethesda, MD). All other reagents used were of analytical grade from Sigma-Aldrich (Madrid, ES, EU). Water was deonized, twice distilled, and passed through Milli-Q equipment (Millipore Iberica, Madrid, ES, EU) to a resistivity better than 18 MΩ cm.

### Sample Preparation

Aliquots containing the appropriate amount of lipid in chloroform/methanol (2∶1 v/v) were placed in a test tube containing the lyophilized peptide, mixed and the solvents removed by evaporation under a stream of O_2_-free nitrogen. Finally traces of solvents were eliminated under vacuum in the dark over night. To obtain multilamellar vesicles, 200 µl of buffer containing 20 mM HEPES, 50 mM NaCl at pH 7.4 was added to the dry mixture. Afterwards, the samples were incubated at 45°C (below the temperature of the lamellar liquid-crystalline to hexagonal-H_II_ transition of pure DEPE), with intermittent vortexing for 30 min to hydrate the samples and obtain multilamellar vesicles (MLV), followed by three freeze–thaw cycles to ensure complete homogenization and maximization of peptide/lipid contacts with occasional vortexing at 45°C. The total lipid concentration was 67 mM for x-ray measurements and 26 mM for DSC. The phospholipid and peptide concentration were measured by methods described previously [40], [41].

### Small-angle X-ray scattering experiments

MLVs at a concentration of 5% (w/w) prepared without or with the peptide at a lipid/peptide molar ratio of 50∶1 were prepared as stated above and submitted to 15 temperature cycles (heating at 45°C and cooling at −20°C). Small angle X-ray diffracction (SAXD) measurements were carried out using a Hecus SWAX-camera (System3, Hecus X-ray Systems, Graz, Austria) as described previously [42] using Ni-filtered Cu-Kα radiation (λ = 1,54 Å) originating from a sealed tube X-ray generator (GE-Seifert, Ahrensburg, Germany) operating at a power of 2 kW (50 kV, 40 mA). Sample-to-detector distance was 27.8 cm. A linear-position-sensitive detector was used with 1024-channel resolution. SAXD angle calibration was done with silver stearate. The measurements were performed with the sample placed in a thin-walled 1-mm diameter quartz capillary held in a steel cuvette holder at different temperatures with an exposure time of 1 h. The SAXD curves were analyzed after background subtraction and normalization in terms of a full q-range model using the program GAP [31].

### Differential scanning calorimetry

MLVs were formed as stated above in 100 mM NaCl, 0.05 mM EDTA and 25 mM HEPES, pH 7.4. The peptides were added to obtain a peptide/lipid molar ratio of 1∶15. The MLV suspension was incubated at 45°C for one hour with occasional vortexing and then centrifuged. The pellets containing lipid vesicles were transferred to 50 µl DSC aluminium pans and hermetically sealed. DSC experiments were performed in a Perkin Elmer Pyris 6 differential scanning calorimeter (Perkin-Elmer Instruments, Shelton, USA) under a constant external pressure of 30 psi in order to avoid bubble formation, and samples were heated at a constant scan rate of 1.5°C/min. In order to avoid artefacts and ensure scan-to-scan reproducibility due to the thermal history of the sample, the first scan was never considered; second and further scans were carried out until a reproducible and reversible pattern was obtained. The last scan was used for calculations. After the thermal measurements, the phospholipid content of the samples was determined. Data acquisition was performed using the Pyris Software for Thermal Analysis, version 4.0 (Perkin-Elmer Instruments LLC) and Microcal Origin 7.0 software (Microcal Software Inc., Northampton, MA, USA) was used for data analysis. The thermograms were defined by the onset and completion temperatures of the transition peaks obtained from heating scans. The phase transition temperature was defined as the temperature at the peak maximum.
